# A nano-Liposomal formulation potentiates antioxidant, anti-inflammatory, and fibrinolytic activities of *Allolobophora caliginosa* coelomic fluid: formulation and characterization

**DOI:** 10.1186/s12896-023-00795-5

**Published:** 2023-08-03

**Authors:** Asmaa E. Farouk, Sohair R. Fahmy, Amel M. Soliman, Sherif Abdelaziz Ibrahim, Shimaa A. Sadek

**Affiliations:** Department of Zoology, Faculty of Science, Cairo University, Giza, 12613 Egypt

**Keywords:** *Allolobophora caliginosa*, Coelomic fluid, Liposomes, Drug delivery system, Stability, Biological activities

## Abstract

**Background:**

Coelomic fluid, a pharmacologically active compound in earthworms, exhibits a range of biological activities, including antioxidant, anti-inflammatory, and anticancer. However, the biological activities exerted by the coelomic fluid can be restrained by its low bioavailability and stability. Liposomes are progressively utilized as an entrapment system for natural bioactive compounds with poor bioavailability and stability, which could be appropriate for coelomic fluid. Thus, the present study was designed to fabricate, characterize, and evaluate the stability of liposomal formulation for *Allolobophora caliginosa* coelomic fluid (ACCF) as a natural antioxidant compound.

**Methods:**

The ACCF-liposomes were developed with a subsequent characterization of their physicochemical attributes. The physical stability, ACCF release behavior, and gastrointestinal stability were evaluated in vitro. The biological activities of ACCF and its liposomal formulation were also determined.

**Results:**

The liposomal formulation of ACCF had a steady characteristic absorption band at 201 nm and a transmittance of 99.20 ± 0.10%. Its average hydrodynamic particle size was 98 nm, with a PDI of 0.29 ± 0.04 and a negative zeta potential (-38.66 ± 0.33mV). TEM further confirmed the formation of vesicular, spherical nano-liposomes with unilamellar configuration. Additionally, a remarkable entrapment efficiency percent (77.58 ± 0.82%) with a permeability rate equal to 3.20 ± 0.31% and a high retention rate (54.16 ± 2.20%) for ACCF-liposomes were observed. The Fourier transform infrared spectroscopy (FTIR) result demonstrated that ACCF successfully entrapped inside liposomes. The ACCF-liposomes exhibited a slow and controlled ACCF release in vitro. Regarding stability studies, the liposomal formulation enhanced the stability of ACCF during storage and at different pH. Furthermore, ACCF-liposomes are highly stable in intestinal digestion conditions comparable to gastric digestion. The current study disclosed that liposomal formulation potentiates the biological activities of ACCF, especially antioxidant, anti-inflammatory, and thrombolytic activities.

**Conclusion:**

These promising results offer a novel approach to increasing the bioaccessibility of ACCF, which may be crucial for the development of pharmaceuticals and nutraceutical-enriched functional foods.

## Introduction

An unhealthy diet accompanies the human lifestyle, daily exposure to a wide range of toxic chemicals, and increased environmental pollution, which may lead to the oxidation of biological molecules [1]. With an increased hectic lifestyle, oxidative damage inevitably increases and may contribute to an increasing burden of chronic diseases, especially cardiovascular diseases, chronic inflammatory diseases, diabetes, and cancer [2, 3]. Consequently, a promising pharmacological approach is urgently required to prevent or treat oxidant-induced cellular and tissue damage [4]. Antioxidant utilization, especially natural antioxidants, is considered an appealing protective strategy to hinder oxidative damage and treat serious impacts on health. Therefore, there is an increasing demand for novel natural antioxidants in the nutraceutical industry owing to the rising consumer prospects worldwide [5, 6]. Earthworm, *Allolobophora caliginosa*, is known as a promising traditional medicine with unbelievable nutritional and health profits [7]. *Allolobophora caliginosa* has a broad spectrum of biological activities such as antioxidant, anti-inflammatory, antibacterial, and antiviral [8]. Moreover, *Allolobophora caliginosa* has specific therapeutic effects, such as reducing cholesterol levels and enhancing bone formation and hepatorenal functions [7, 9, 10]. The biological and therapeutic activities of *Allolobophora caliginosa* are mainly exerted by its coelomic fluid content of phenolic compounds and flavonoids, especially gallic acid and quercetin, which have potent antioxidant activity [8]. Additionally, the coelomic fluid of *Allolobophora caliginosa* contains antioxidant amino acids, including alanine, valine, leucine, and isoleucine [8]. Several previous studies demonstrated that the coelomic fluid of *Allolobophora caliginosa* exerts various therapeutic potentials, especially alleviating pain, reducing hyperthermia, amelioration of hepatorenal toxicity, and regulating osteoblastic/osteoclastic remodeling [8–10]. Due to the nutritional and health benefits of coelomic fluid, it can be a good source of natural antioxidants in the healthcare and nutraceuticals industry for specific dietary applications.

However, the advantages offered by natural antioxidant compounds can be restrained by their low bioavailability, stability, and the loss of the bioactivity of active ingredients in biological systems and subsequently, reduce their free radical scavenging properties [11]. Thus, entrapment technology can effectively overcome these challenging properties by enhancing bioavailability, avoiding the degradation and oxidation of sensible compounds, increasing the stability of these molecules, and maintaining their antioxidant activity [12]. Liposomes are one of the most effective entrapment technologies that gained more attention for preserving natural antioxidant compounds and enhancing their efficacy in scavenging free radicals [13]. Moreover, Suntres [4] reported that the entrapment of antioxidant molecules in liposomes improves their potential against oxidant-induced tissue damage because of liposomes’ ability to facilitate intracellular delivery and increase the retention period of entrapped antioxidants inside the cell.

Liposomes are spherical natural phospholipid vesicles comprising one or more concentric lipid bilayers enclosing discrete aqueous spaces. This structure provides their amphipathic behavior, allowing the entrapment of both lipophilic and hydrophilic compounds [14]. The liposomal entrapment system represents the prospect vectors of antioxidant molecules because liposomes provide many advantages, including high stability in the biological system, controlling the release rate of targeted entrapped contents, and possessing a composition compatible with the human body [15]. Additionally, this entrapment system can improve the performance of the encapsulated antioxidant molecules, especially their bioavailability, half-life, and selective and targeted delivery [16]. Recently, the encapsulation of natural antioxidants was the focus of several studies, and there is no comprehensive study regarding the encapsulation of *Allolobophora caliginosa* coelomic fluid into liposomes. Therefore, the present study was intended to develop, characterize, and evaluate the stability of liposomal formulation containing *Allolobophora caliginosa* coelomic fluid (ACCF) as a natural antioxidant compound. Additionally, the release behavior of ACCF from liposomes was evaluated in vitro, and an in vitro dynamic gastrointestinal model was used to assess the behavior of liposomes containing ACCF during the gastrointestinal tract. Then, the various biological activities, such as antioxidant, anti-inflammatory, and fibrinolytic, were evaluated in vitro for free ACCF and ACCF entrapped in liposomes.

## Materials and methods

### Chemicals and reagents

The phospholipid ingredient of liposomes (soybean lecithin, 98.7% purity) was provided by Alpha Chemika (Mumbai, India). Cholesterol, 1,1-Diphenyl-2-picrylhydrazyl (DPPH), Griess reagent, pepsin (3000U/mg), pancreatin, and the salts used for preparing simulated gastric and intestinal fluids were purchased from Sigma Aldrich (St Louis, Missouri, USA).

### *Allolobophora caliginosa* collection

Healthy adult earthworms, *Allolobophora caliginosa*, were obtained from a profitable Vermin culture at the Giza Governorate, Egypt. They were preserved in a plastic container with decomposed organic substances until transported to the laboratory. Before collecting coelomic fluid, the earthworms were cleaned using running tap water and phosphate buffer saline (PBS; 0.01 M, pH 6.5) to remove any undesirable matters attached to their body surface and then placed on a moist filter paper for 24 h to allow the draining of their gut, preventing any contamination during the coelomic fluid collection.

### *Allolobophora caliginosa* coelomic fluid (ACCF) collection

Coelomic fluid was directly collected from the body cavity of earthworms without causing any harm by using the heat shock method [17, 18]. In brief, three to four healthy earthworms weighing 0.8–1.2 g were placed in a clean Petri dish and exposed to heat shock using hot water (45–50 °C) in a glass beaker. As a result of heat shock drips, the ACCF was extruded via the dorsal epidermal pore into the media and collected at the lower side of the Petri plate. Next, the extracted fluid was assembled in falcon tubes using a sterilized pipette with a fine nozzle. Afterward, the coelomic fluid was centrifuged using a centrifuge (KUBOTA 2100, Japan) at 4000 RPM for 30 min at 4 °C. Finally, the resultant supernatant was concentrated and dried using a lyophilizer (EDWARDS, Italy).

### Preparation of liposome entrapping *Allolobophora caliginosa* coelomic fluid (ACCF-liposomes)

The lipid film hydration method was used to prepare a liposomal formulation containing ACCF [19]. Briefly, 225 mg of purified soy lecithin and 25 mg of cholesterol were dissolved in dichloromethane (6 ml) to form an organic solvent mixture. Then, 45 mg of ACCF was added to the mixture, and the resulting solution was dried into a thin lipid film using a rotary evaporator (YR02306, Kalstein, France) at 45 °C under reduced pressure. The dry thin film was hydrated with PBS (pH 7.4) and shaken vigorously for 2–3 min. Next, the liposome was formed and sonicated using a Sonics Vibra-Cell sonicator, USA, for 5 min. at a measured power of 120 W in an ice bath to ensure complete dispersion. It was then stored at 4 °C until used.

### Physicochemical characterization of ACCF-liposomes

#### Particle size and zeta potential analysis

To evaluate the stability of ACCF-liposomes, their average hydrodynamic size, polydispersity index (PDI), and zeta potential were measured using dynamic light scattering (DLS) with a Zetasizer Nano ZS (Malvern Instruments Ltd, USA) [20]. The liposome samples were first diluted with 0.2 M phosphate buffer (pH 7.0) to prevent particle aggregation before being placed in a standard capillary electrophoresis cell with gold electrodes. The resulting data provided information on the average hydrodynamic size, PDI, and zeta potential of the ACCF-liposomes.

#### Morphological characterization of ACCF- liposomes analysis

A morphological surface of the prepared ACCF-liposomes was accomplished using a transmission electron microscope (TEM; JEOL, JEM-1400TEM, USA) with the negative stain method [21]. Briefly, 20 μl of the ACCF-liposome sample was applied to a copper grid coated with carbon film for 10 min, and the excess fluid was eliminated using filter paper. Then, the grid was stained with 3% phosphotungstic acid and permitted to dry for 3 min. After being negatively stained and air-dried at room temperature, the microstructure of ACCF liposomes was precisely investigated. Micrographs were captured using an acceleration voltage of approximately 200kV.

### Structural characterization of ACCF-liposomes

#### Fourier transform infrared (FT-IR) analysis

To assess any chemical reactions between ACCF and other ingredients, Fourier transform infrared spectroscopy (FT-IR) was used to analyze the functional groups of ACCF liposomes. The process involved freeze-drying the ACCF-liposomes, mixing them with potassium bromide (KBr) pellet, and pressing them to form disks [22]. IR spectra (range 4000–400 cmˉ1) were then recorded using an FT-IR spectrophotometer (JASCO FTIR-6200, JASCO International Co., Ltd- Japan).

#### X-ray diffraction study

X-ray diffraction (XRD) was performed to ascertain the purity and crystalline structure of ACCF-liposomes using an X-ray diffractometer (1.5426 Å, Reguka Miniflex, India) with Cu Kα radiation, operating at 30 kV/15 mA. All measurements were achieved at room temperature within the diffraction angle 2*θ* range of 0–80° and at a speed of 1° per min. Also, XRD was used to estimate the average crystallite size of ACCF-liposome using Debye–Scherrer’s formula [23]:

$$\mathrm{D}=0.9\uplambda /\mathrm{\beta cos \theta }$$

D represents crystallite size; λ = 0.154 nm; β represents full width at half maximum of peaks in radian at any 2θ in the pattern.

#### Assessment of optical absorption of ACCF-liposomes

The optical absorption of ACCF-Liposome was determined using a double-beam ultraviolet–visible (UV–Vis) near-infrared (NIR) spectrophotometer (Cary 5000, Varian, Palo Alto, California, USA) at wavelengths ranging from 200 to 800 nm at room temperature.

#### Transmittance percentage determination of ACCF-liposomes

The percentage of transmittance of the ACCF-liposomes was analyzed using the spectrophotometric method described by Laxmi et al. [24]. The analysis was conducted at 650 nm using a U-2001 model 121–0032 Hitachi spectrophotometer compared to distilled water as the blank.

#### Determination of entrapment efficiency percent (%)

The efficiency of liposome to entrap ACCF was determined using the ultracentrifugation method based on the approach adopted by Bendas and Tadros [25]. Concisely, ACCF-liposome samples (2 mL) were centrifuged using a centrifuge (KUBOTA 2100, Japan) at 12,000 rpm for 30 min to separate the free ACCF from the encapsulated one. Then, the clear supernatant that contained free ACCF was collected and vortexed to obtain a homogeneous solution. Afterward, the absorbance of the free ACCF in the supernatant was determined using a UV–visible spectrophotometer (U-2001, model 121–0032 Hitachi, Tokyo, Japan) at 360 nm (the resonance absorption of ACCF). To determine the concentration of free ACCF in the supernatant, the absorbance of ACCF was measured at different concentrations spectrophotometrically at 360 nm. Then, the standard curve of ACCF was accomplished by plotting the absorbance against its different concentrations. The entrapment efficiency percent of liposome was calculated from the following equation:

$$\mathrm{Entrapment efficiency percent }\left(\mathrm{\%}\right)=\frac{\mathrm{Total ACCF concentration}-\mathrm{free ACCF concentration}}{\mathrm{Total ACCF concentration }}\times 100$$

Additionally, the retention rate was calculated according to the following formula [26]:

$$\mathrm{Retention rate}=\frac{Encapsulated amount of ACCF after storage}{Encapsulated amount of ACCF intially prepared}\times 100$$

#### In vitro ACCF release study

The release of ACCF from liposomal formulation was evaluated in vitro using a dialysis method as Salem et al.[27] described. Firstly, a cellulose acetate dialysis bag (Spectra/ Por, MW cutoff 12,000, Spectrum, Canada) was soaked before PBS at room temperature for 12 h to remove the preservative, then carefully rinsing it in distilled water. Then, 3 mL of ACCF-liposomes was pipetted into a dialysis bag and immersed in 100 mL of PBS (pH 7.4). The buffer containing the dialysis bag was magnetically stirred at 150 rpm. After that, 2 mL of the immersing solution was withdrawn at several time intervals (each one hour), and the immersing solution was immediately substituted with equal volumes of fresh PBS. The absorbance of the obtaining immersing samples was measured at 360 nm using a spectrophotometer (U-2001, model 121–0032 Hitachi, Tokyo, Japan), and then, the % release of ACCF was calculated as previously described. The experiment was stopped when the ACCF concentration in the immersing medium became steady. Similarly, a release of free ACCF at a concentration similar to the encapsulated liposomal ACCF was carried out using the same dialysis bag method.

### Stability studies of ACCF-liposomes

#### The physical stability study

The physical stability of ACCF-liposomes was evaluated by monitoring the changes in particle size, zeta potential, and PDI during storage at 4°C for one month. Furthermore, the capability of the prepared liposome formulation to maintain ACCF within the liposomes during the storage period was evaluated by determining the entrapment efficiency percent (%) as previously described [25]. Also, the permeability of liposomes entrapping ACCF was calculated after one month of storage at 4°C using the following equation [28]:

$$\mathrm{Permeability rate}=\frac{InitialEE-EE after 30 day}{initial EE}\times 10$$

where EE is the entrapment efficiency percent (%).

#### The effect of pH on ACCF- liposomes stability

The stability of prepared ACCF-liposomes was evaluated at varying pH values, as Aisha et al. [29] illustrated. Briefly, a series of ACCF-liposome samples were diluted at a ratio 1:4 in phosphate buffer saline (PBS) at different pH values (1.5, 4.5, 6.8, 7.4) and water. Then, the samples were incubated overnight at 37°C for 16h. Subsequently, the diluted ACCF-liposome solutions were centrifuged at 8000 rpm at 25°C for 10 min. Then the concentration of ACCF in the supernatant was determined by UV–Vis spectrophotometry (U-2001, model 121–0032 Hitachi, Tokyo, Japan) at 360 nm. The results are exhibited as a percentage of soluble fraction relative to liposomes diluted in water.

### Assessment of in vitro gastrointestinal behavior of ACCF-liposomes

#### Preparation of simulated gastric and intestinal fluids

Simulated gastric fluid (SGF) and simulated intestinal fluid (SIF) were prepared as previously designated by Singh and Sarkar [30]. The SGF was prepared by dissolving 2 g NaCl and 1.4 g pepsin (3000 U/mg) in distilled water and adding 7 mL of Conc. HCl (1M). Then, the solution was diluted to obtain 1L of the SGF, and the pH was adjusted to 1.2. On the other hand, the SIF contained 6.8 g of dipotassium phosphate and 190 mL of 0.1 M NaOH, with 150 mM NaCl and 30 mM CaCl_2_. Then, the prepared mixture was diluted to 1 L, and its pH was adjusted to 7.4. The bile salt and pancreatin were added to the prepared SIF at 0.2 mg/ml and 0.032 mg/ml, respectively. SGF and SIF were incubated at 37°C with continuous shaking before in vitro liposomal digestion.

#### Stability of ACCF-liposomes during in vitro digestion

The simulated gastric and intestinal model was used to characterize the potential gastrointestinal (GI) behavior of the ACCF-liposomes according to the method described by Malinauskyte et al. [31] with slight modification. The ACCF-liposomes were mixed with SGF or SIF in the ratio of 1:3 and incubated at 37°C for 2h, representing the average duration of GI transit. During digestion, a desired volume of the mixture was withdrawn at several time intervals (0–120 min) for testing the release rate of ACCF via determining entrapment efficiency percent (%), as described previously.

### Evaluation of biological activities of ACCF and ACCF-liposomes

#### The antioxidant potency of ACCF and ACCF-liposomes

##### Evaluation of total antioxidant capacity

The phosphomolybdenum assay was used to evaluate the total antioxidant capacity of ACCF and ACCF-liposomes according to the method illustrated by Prieto et al. [32]. Briefly, 0.1 ml of ACCF and ACCF-liposomes at various concentrations (100–500 µg/ml DMSO) was added to 1 ml of molybdate reagent solution, which consisted of 0.6 M H_2_SO_4_, 28 mM sodium phosphate, and 4 mM ammonium molybdate. Subsequently, the mixture was incubated at 95 °C for 90 min. Then, the reaction tubes were cooled, and the absorbance of the mixture was measured at 695 nm using a UV–Vis spectrophotometer (U-2001, model 121–0032 Hitachi, Tokyo, Japan) against blank (0.1 ml DMSO and 1 ml of molybdate reagent solution). The total antioxidant capacity was expressed as the number of gram equivalent of ascorbic acid.

##### Evaluation of free radical scavenging potency

A) DPPH radical scavenging assay

The free radical scavenging potency of ACCF and ACCF-liposomes was evaluated using DPPH assay as Brand et al.[33] described. Firstly, the various concentrations ranging from 50-500 μg/ml of ACCF, ACCF-liposomes, and ascorbic acid (standard antioxidant) were prepared. Subsequently, 2ml of ACCF, ACCF-liposomes, or ascorbic acid at different concentrations were mixed with 2ml of DPPH at a concentration of 0.1mM. Then, all tubes were shaken and incubated at 37°C for 30 min. The control tube (DPPH only) was prepared in the same manner. The absorbance of each solution was measured at 517 nm against methanol as the blank. The DPPH radical scavenging activity (%) was calculated using the following equation:

$$\mathrm{DPPH radical scavenging activity }(\mathrm{\%})=\left[\left({\mathrm{A}}_{\mathrm{control}}-{\mathrm{A}}_{\mathrm{sample}/\mathrm{standard}}/{\mathrm{A}}_{\mathrm{control}}\right)\right]\times 100$$

Also, half-maximal inhibitory concentration (IC_50_) was calculated based on an equation from the plot graph between the percentage of DPPH radical scavenging activity and the various sample concentrations.

b) Nitric oxide radical scavenging assay

Nitric oxide (NO) radical scavenging potency of ACCF and ACCF -liposomes was determined using the Griess Illosvoy reaction as previously described with slight modifications [34]. Briefly, 2 ml of 10mM sodium nitroprusside and 0.5 ml of phosphate buffer saline (pH 7.4) were mixed with 0.5 ml of ACCF, ACCF-liposomes, and Ascorbic acid at various concentrations. Then, the reaction mixture was incubated at 25°C for 3h to generate nitric oxide radicals interacting with oxygen and producing the nitrite ion. Subsequently, the formed nitrite ion was assayed by adding 1 ml of Griess reagent to an equal volume of the reaction mixture and incubated at 25°C for 30 min. The absorbance of the chromophore (purple azo dye) formed during the Griess reaction was measured at 546 nm. The NO radical scavenging activity (%) was calculated using the following equation:

$$\mathrm{NO radical scavenging activity }(\mathrm{\%})= \left[\left({\mathrm{A}}_{\mathrm{control}}-{\mathrm{A}}_{\mathrm{sample}/\mathrm{standard}}/{\mathrm{A}}_{\mathrm{control}}\right)\right]\times 100$$

Also, half-maximal inhibitory concentration (IC_50_) was calculated as described previously in the DPPH assay.

##### Evaluation of anti-inflammatory potency of ACCF and ACCF-liposomes

The anti-inflammatory potency of ACCF and ACCF- liposomes was evaluated using in vitro human red blood cell (HRBC) membrane stabilization assay, as Gandhisan et al. described [35]. The anti-inflammatory potency of ACCF and ACCF-liposomes was compared with Aspirin as a standard anti-inflammatory drug at different concentrations (50–400 μg/ml). Firstly, human red blood cell suspension (HRBC) was constituted with normal saline by collecting fresh whole human blood from a healthy volunteer in a heparinized tube and then centrifuged at 3000 rpm for 10 min. Then, the packed red blood cells were diluted with saline to reconstitute a 10% HRBC suspension. In each tube, 1 ml of ACCF, ACCF-liposomes, or aspirin at different concentrations was mixed with 1 ml of HRBC suspension, and all tubes were subsequently incubated at 56ºC for 30 min. Following incubation, the tubes were centrifuged at 2500 rpm for 5 min. The hemoglobin content in the supernatant was measured using a UV–Vis spectrophotometer (U-2001, model 121–0032 Hitachi, Tokyo, Japan) at 560 nm. The control tube containing HRBC suspension and normal saline solution which tested in the same manner. The percentage of HRBCs membrane stabilization was calculated using the following equation:

$$\mathrm{\% HRBCs membrane stabilization }= 100-\left[\left({\mathrm{A}}_{\mathrm{sample or standard}}/{\mathrm{A}}_{\mathrm{control}}\right)\times 100\right]$$

Then, half-maximal inhibitory concentration (IC_50_) was calculated based on an equation from the plot graph between the percentage of HRBCs membrane stabilization and the various sample concentrations.

##### Evaluation of fibrinolytic potency of ACCF and ACCF-liposomes

As reported earlier, the clot lysis assay was carried out to evaluate the fibrinolytic potency of ACCF and ACCF-liposomes [36]. Briefly, 4 ml venous blood was drawn from a healthy volunteer and immediately distributed in pre-weighed sterile micro-centrifuge tubes (0.5 ml/tube). Then, all micro-centrifuge tubes were incubated at 37°C for 45 min to permit clot formation. Once the clot was formed, serum was wholly removed without disturbing the clot, and then, each tube was re-weighed again to calculate the clot formed weight (clot weight = weight of clot containing tube –weight of empty tube). Next, 100 μl of ACCF, ACCF- liposomes, or streptokinase (as standard fibrinolytic drug) were added separately to different micro-centrifuge tubes containing clots. Meanwhile, the control tube containing 100 μl of distilled water and the formed clot. All the tubes were incubated at 37°C for 90 min, and then, the resulting fluid was aspirated carefully, and tubes were again weighed to observe the difference in weight after clot disruption. The difference obtained in weight taken before and after clot lysis was expressed as a percentage of clot lysis.

### Statistical analysis

All data are expressed as mean ± standard error (SEM) of three parallel sample measurements. Significant differences between the means were determined by one-way analysis of variance (ANOVA) with the Duncan post hoc test using SPSS (SPSS Inc., Chicago, IL, USA) software. But significant differences between means of storage stability data were evaluated by Student's t-test at a 95% confidence level. P values < 0.05 were regarded as statistically significant.

## Results

### Physicochemical characterization of ACCF-liposomes

#### Average hydrodynamic diameter, zeta potential, and PDI

**Fig.1:**
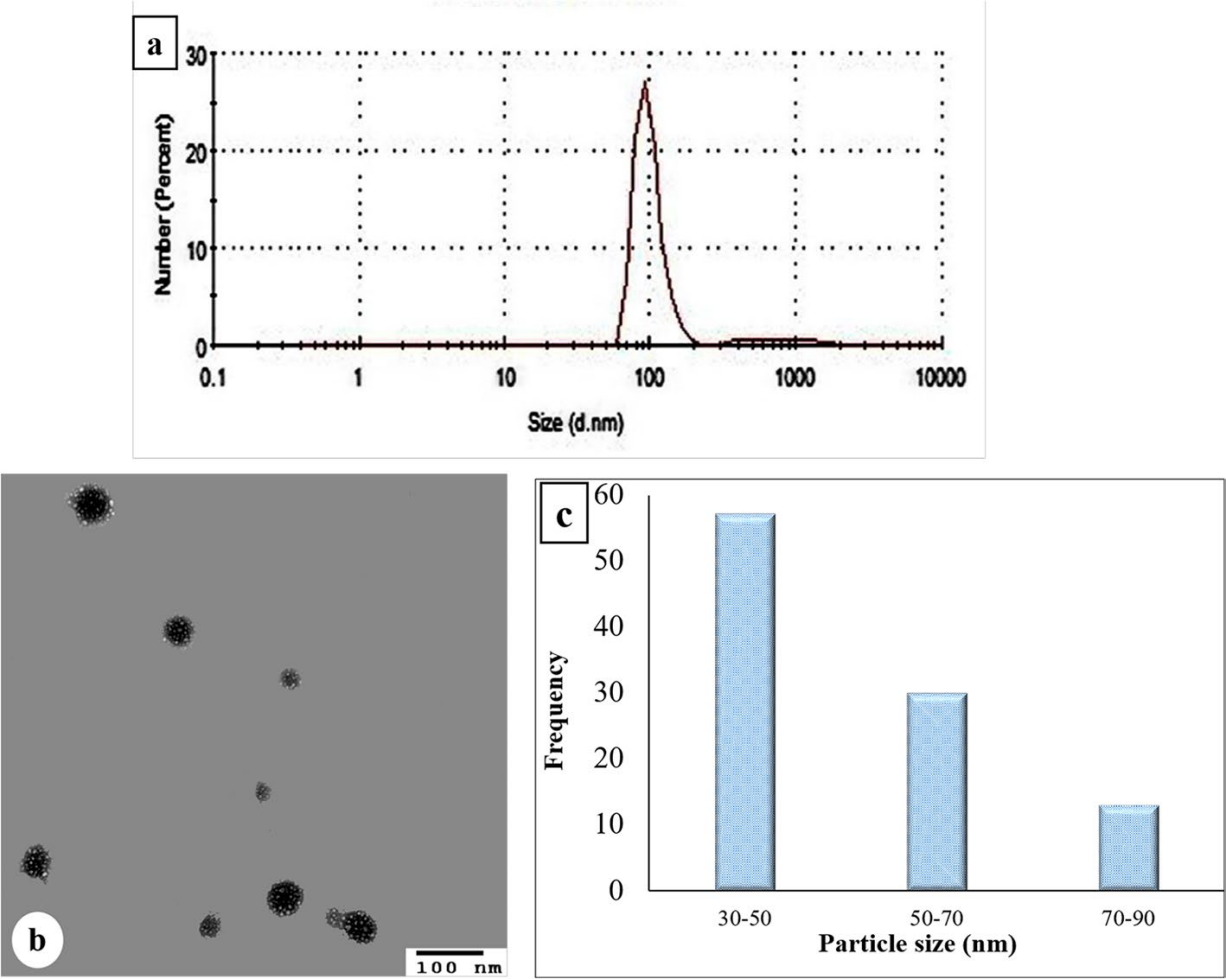
Physicochemical characterization of ACCF-liposomes using dynamic light scattering technique and a transmission electron microscope (TEM). (**a**) Particle size distribution of ACCF-liposomes using dynamic light scattering technique demonstrating an apparent particle size of 98 nm. (**b**) TEM image showing the spherical shape of ACCF-liposomes. (**c**) The size distribution histogram generated by TEM reveals an apparent particle size of 40 nm. ACCF: *Allolobophora caliginosa* coelomic fluid

Figure 1a and Table 1 demonstrate that the average hydrodynamic size of liposomes entrapped ACCF was 98 nm with a PDI of 0.29±0.04, confirming the nanometric size of the liposomal formulation with homogeneous dispersion. Concerning the surface electric charge, the zeta potential of ACCF-liposomes was -38.66±0.33mV, illustrating their excellent stability (Table 1).

**Table1:** Physicochemical properties of ACCF-liposomes

Physicochemical attribute	ACCF-liposomes
PDI	0.29 ± 0.04
Zeta potential (mv)	-38.66 ± 0.33
%Transmittance	99.20 ± 0.10
Entrapment efficiency percent (%)	77.58 ± 0.82
Retention rate (%)	54.16 ± 2.20

Values are means of three replicate determinations ± SEM

*ACCF Allolobophora caliginosa* coelomic fluid, *PDI* Polydispersity index

### The morphological surface of ACCF-liposomes

TEM photograph of the liposomes entrapped ACCF confirmed the formation of vesicular, spherical nano-liposomes with unilamellar configuration (common features of nano-liposome) (Fig. 1b). Furthermore, the TEM study confirmed that the liposomal particles are monodisperse and nanosized with an average size of approximately 40 nm, as shown in Fig. 1c

### Structural characterization of ACCF-liposomes

#### FT-IR Spectra

**Fig. 2:**
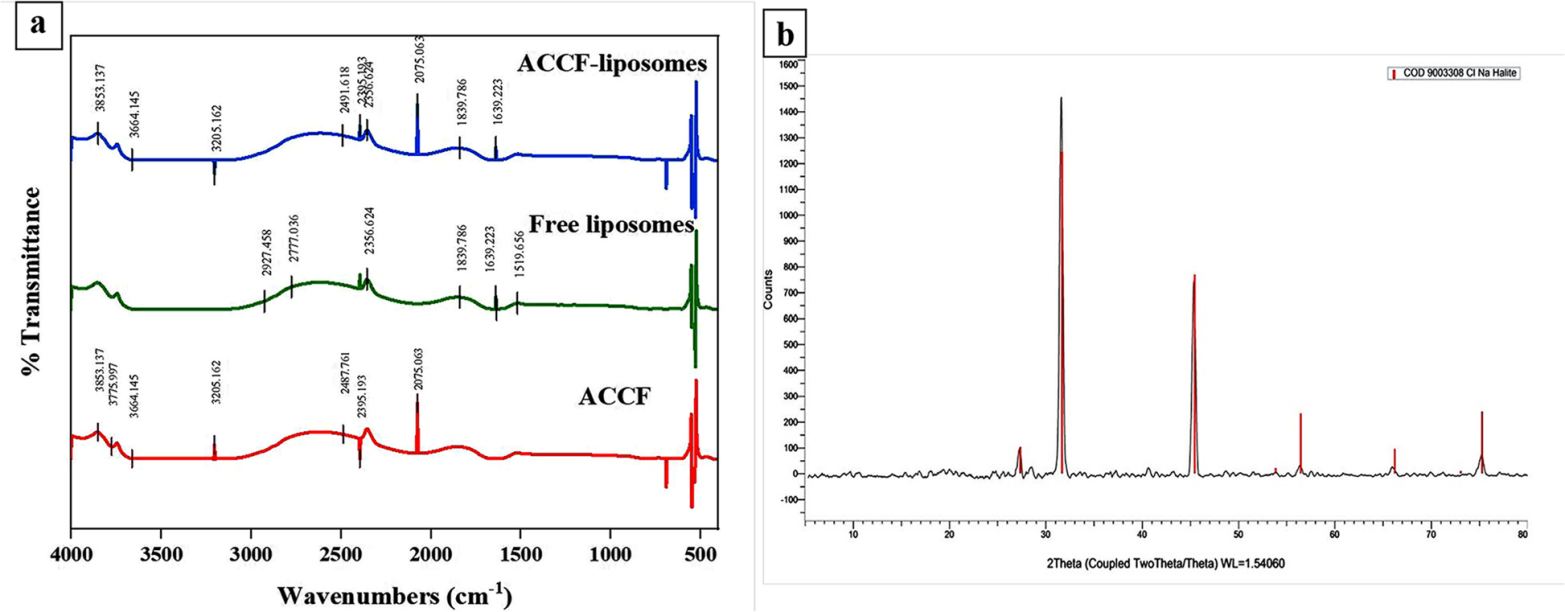
Structural characterization of ACCF-liposomes using Fourier transform infrared (FT-IR) and X-ray diffraction (XRD) analysis. (**a**) FT-IR spectra of ACCF, free liposomes, and ACCF-liposomes. (**b**) X-ray diffraction pattern demonstrating crystalline structure and the size of ACCF-liposomes. ACCF: *Allolobophora caliginosa* coelomic fluid

To study and evaluate the existence of different interactions between the phospholipid of liposomes and ACCF, FT-IR spectra of ACCF, free liposomes, and ACCF- liposomes were studied (Fig. 2a). The FT-IR spectrum of ACCF indicated the presence of OH, C–H, C–O, C–C, C≡C chemical groups with characteristic bands at 3853.13, 3775.99, 3664.14, 3204.14, 2487.76, 2395.19, and 2075.063, respectively. The FT-IR spectrum of free liposomes showed bands at 2927.45, 2777.04, 2356.62, 1839.78, 1639.23, and 1519.65. However, the FT-IR spectrum of liposomes entrapped ACCF showed no appearance or disappearance of absorption peaks compared to ACCF and free liposome spectra, indicating that the encapsulation of ACCF within the formulated liposomes did not develop any covalent bonds (Fig. 2a).

#### XRD study

The XRD diffractogram showed that ACCF-liposomes had distinct and narrow peaks ranging from 27° to 80°, with the highest peak at 2θ = 31.70°, indicating their crystalline nature (Fig. 2b). Moreover, Scherrer’s formula was used to calculate the average crystallite size of ACCF-liposomes, which was found to be 21.62 nm.

#### Optical absorption and % transmittance of ACCF-liposomes

**Fig. 3:**
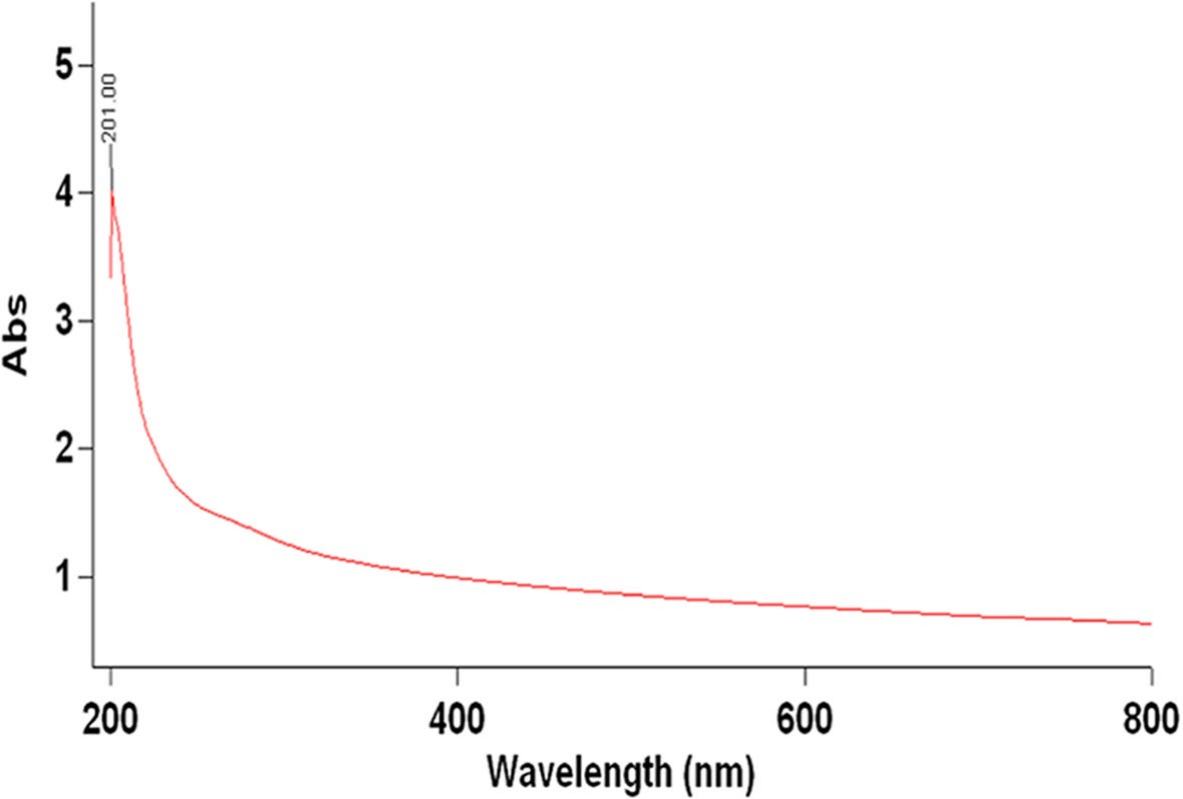
UV–visible absorption spectra of ACCF-liposomes showing the evolution of a steady absorption peak at 201 nm. ACCF: *Allolobophora caliginosa* coelomic fluid

The UV–Vis spectrophotometer revealed a steady characteristic absorption band for ACCF-liposomes at 201 nm, as demonstrated in Fig. 3. Moreover, the transmittance percentage of ACCF-liposomes was 99.20 ± 0.10%, suggesting transparency of liposomal formulation of ACCF (Table 1).

#### Entrapment efficiency percent (%) and retention rate

As shown in Table 1, liposomes had a greater ability to entrap ACCF with an entrapment efficiency of 77.58 ± 0.82%. The stability of ACCF-liposomes was also confirmed by their retention rate reaching 54.16 ± 2.20% after 30 days (Table 1).

#### In vitro ACCF released from liposomes

**Fig. 4:**
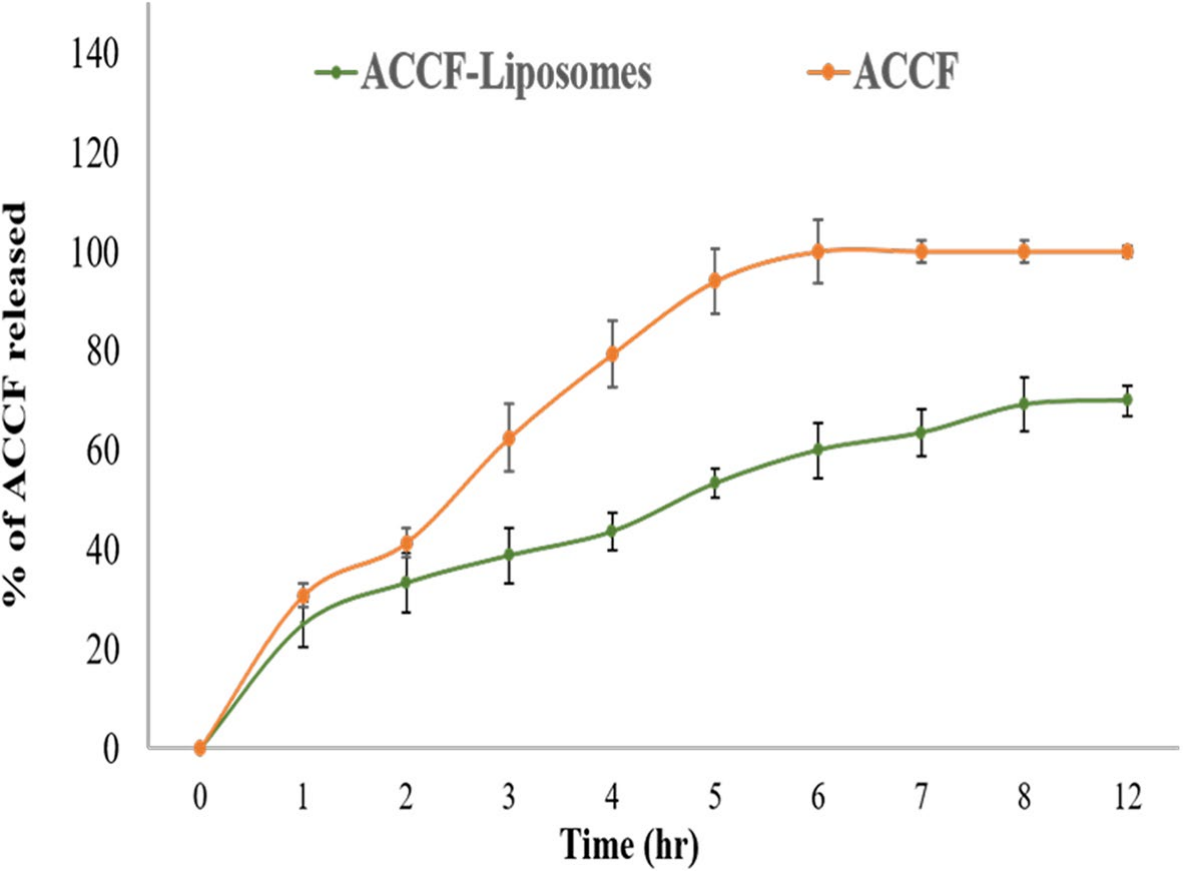
The in vitro release behavior of ACCF and ACCF-liposomes prepared by lipid film hydration method. Values are the mean of three replicate determinations ± SEM. ACCF: *Allolobophora caliginosa* coelomic fluid

The liposomes entrapped ACCF exhibited an initial burst release of 25% within 2h of diffusion (Fig. 4). Subsequently, sustained ACCF release up to 70% was noticed between 2 to 8 h. The remaining 30% of ACCF-liposomes might be constantly trapped inside the liposomes, suggesting the liposomal release in a bi-phasic fashion, *i.e.*, an initial fast drug diffusion followed by slow release through the membrane. Meanwhile, free ACCF diffused rapidly, where almost 60% of it diffused after 3h. After 5 h, 94% of the free ACCF diffused through the dialysis membrane, indicating that liposomal encapsulation enables slow release up to 8 h (Fig. 4).

### Stability of ACCF-liposomes

#### Storage stability of ACCF-liposomes

As shown in Table 2, the physical stability study of ACCF-liposomes implied their colloidal system stability as indicated by a non-significant change in their mean hydrodynamic diameter, zeta potential, PDI, entrapment efficiency percent (%) after storage at 4°C for one month. Furthermore, the permeability rate of ACCF-liposomes remained stable after 30 days of storage at 4°C, with a rate of 3.20 ± 0.31% (Table 2). This suggests that the liposomes are highly stable.

**Table 2:** Storage stability of ACCF-liposomes at 4°C

Sample	ACCF-liposomes	
	**Before**	**After 30 days**
Zeta potential (mv)	38.66 ± 0.33	37.66 ± 0.33
PDI	0.29 ± 0.01	0.31 ± 0.01
Mean particle size (nm)	98.00 ± 0.33	97.00 ± 0.01
Entrapment efficiency percent (%)	77.58 ± 0.82	75.48 ± 0.29
Permeability rate (%)	–-	3.20 ± 0.31

Values are means of three replicate determinations ± SEM

ACCF: *Allolobophora caliginosa* coelomic fluid; PDI: polydispersity index

#### pH stability of ACCF-liposomes

The stability of the ACCF-liposomes at different pH 1.6, 5.5, and 7.4 was evaluated to forecast their stability in the digestive tract, as demonstrated in Table 3. Slight accumulation and precipitation of ACCF-liposomes arose instantly after mixing with PBS at pH 1.5 but mixing with PBS at pH 4.5, 6.8, and 7.4 or water, neither agglomeration nor precipitation was noticed. This is revealed by the percentage of the soluble fraction relative to that in water was 73.69 ± 0.46% (pH 1.6), 89.80 ± 0.02% (pH 4.5), 94.87 ± 0.04% (pH 6.8) and 99.36 ± 0.08% (pH 7.4). Notably, ACCF- liposomes are highly stable at pH 4.5, 6.8, and 7.4, comparable to their stability at pH 1.5, implying their stability in biological fluids at physiological pH.

**Table 3:** Stability of ACCF-liposomes at different pH

Sample	pH value			
	**1.5**	**4.5**	**6.8**	**7.4**
ACCF-liposomes	73.69 ± 0.46^a^	89.81 ± 0.11^b^	94.87 ± 0.37^c^	99.36 ± 0.83^d^

Values are means of three replicate determinations ± SEM

Values with different superscript letters are significantly different (P < 0.05)

*ACCF Allolobophora caliginosa* coelomic fluid

#### In vitro gastrointestinal behavior of ACCF-liposomes

**Fig. 5:**
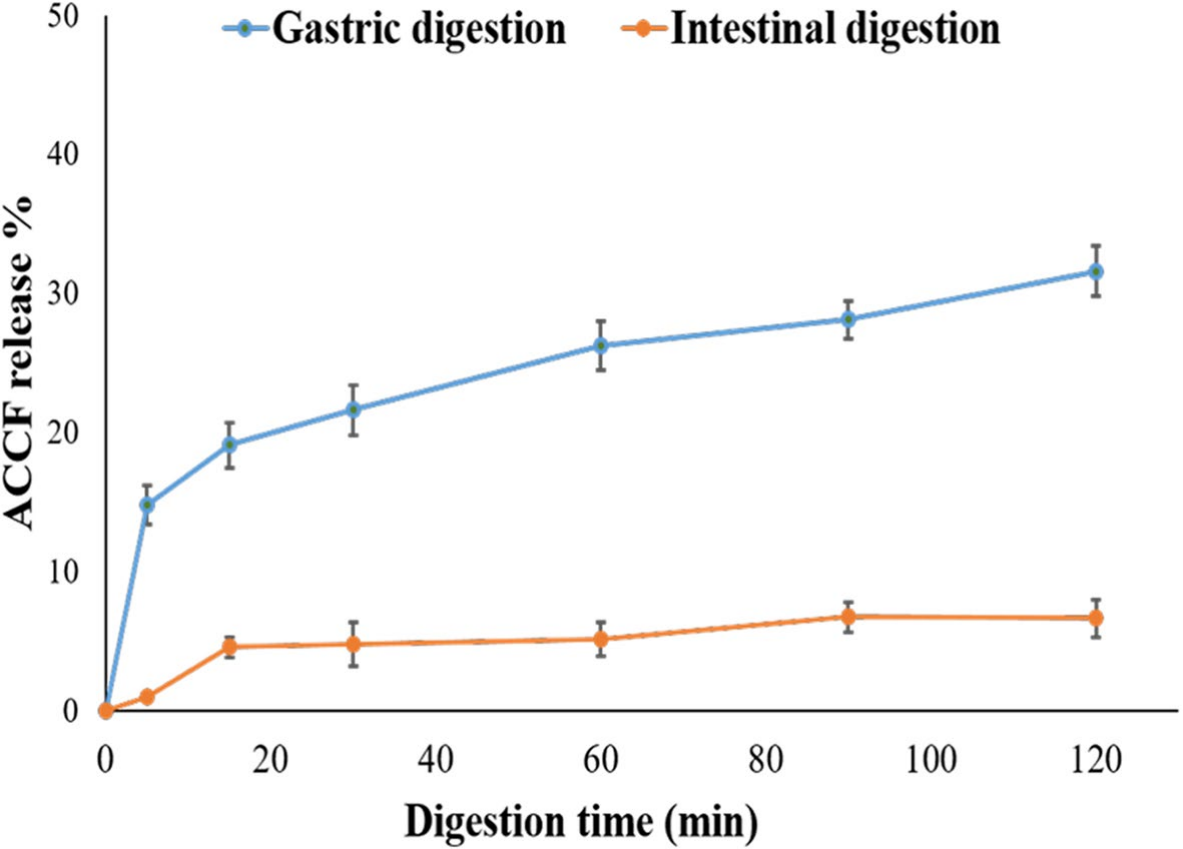
Stability of ACCF-liposomes during in vitro simulated gastrointestinal digestion. Values are the mean of three replicate determinations ± SEM. ACCF: *Allolobophora caliginosa* coelomic fluid

Figure 5 demonstrates that the release rate of ACCF-liposomes increased gradually with the increase of digestion time during in vitro gastric and intestinal digestion. During the first 30 min, the release of the ACCF from the liposomes during simulated gastric digestion was more rapid than its release during intestinal digestion, with a release rate of 21.60 ± 1.89% and 4.73 ± 0.60%, respectively. After 120 min of digestion, 67.25% of ACCF was retained in the liposomes during the simulated gastric digestion process but over 93% of ACCF was still retained in the liposomes during the simulated intestinal digestion process, indicating that ACCF-liposomes are highly stable in intestinal digestion conditions comparable to their stability in gastric digestion.

### Biological activities of ACCF-liposomes

#### The antioxidant potency of ACCF and ACCF-liposomes

##### Total antioxidant capacity

**Fig. 6:**
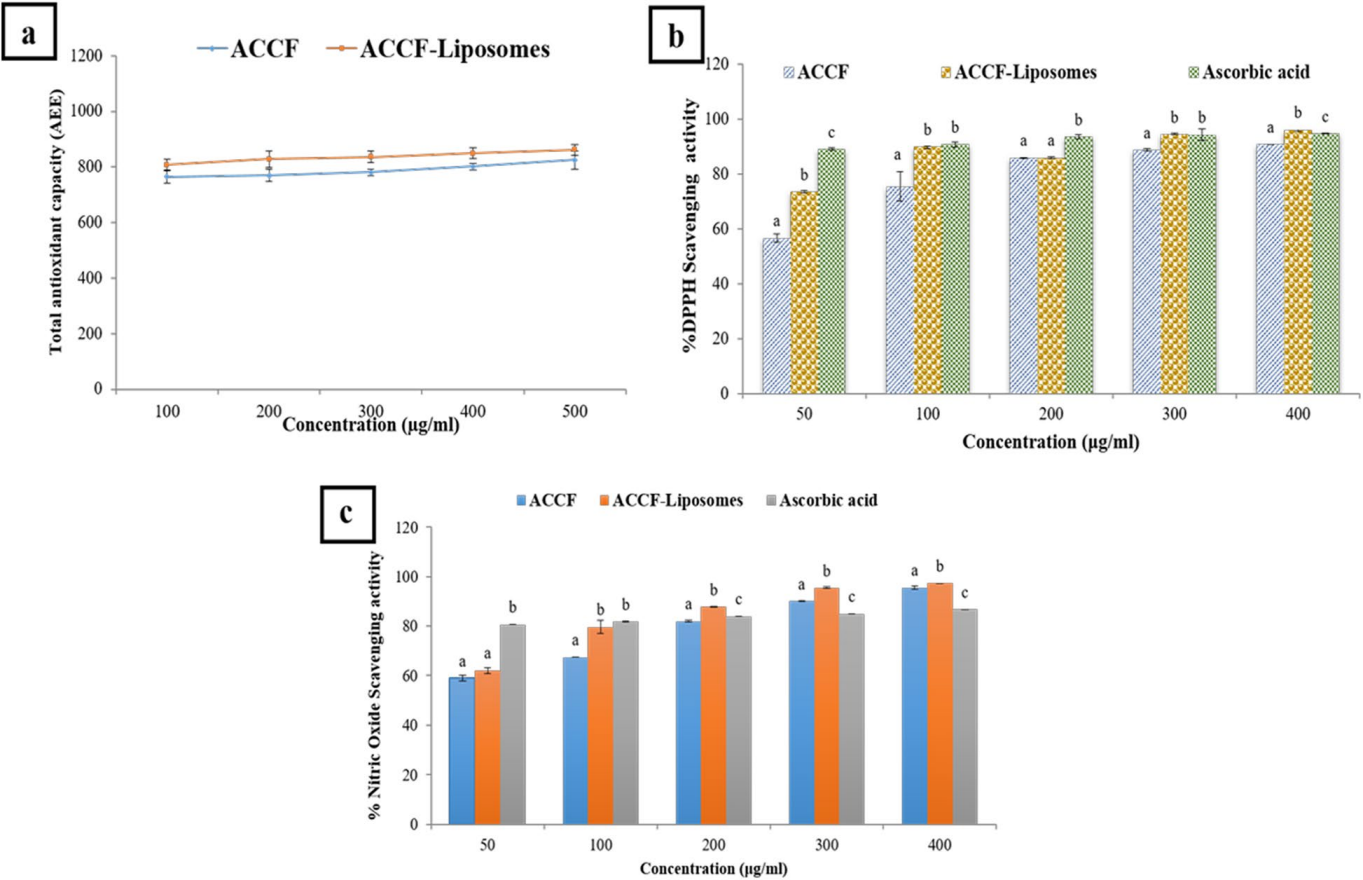
Antioxidant potency of ACCF and ACCF-liposomes. (**a**)Total antioxidant capacity of ACCF and ACCF-liposomes; (**b**) DPPH radical scavenging activity of ACCF and ACCF-liposomes; (**c**) Nitric oxide radical scavenging activity of ACCF and ACCF-liposomes. Values are the mean of three replicate determinations ± SEM. Values with different superscript letters are significantly different (P < 0.05) for each concentration. ACCF: Allolobophora caliginosa coelomic fluid. DPPH: 1,1-Diphenyl-2-picrylhydrazyl

The liposomes containing ACCF showed significant antioxidant activity, which was similar to that of free ACCF in all concentrations, as shown in Fig. 6a. ACCF-liposomes exerted substantial total antioxidant capacity equivalent to 807.65 ± 20.01 mg/g ascorbic acid at a concentration of 100 µg/ml. In contrast, the total antioxidant capacity of ACCF was equivalent to 765.37 ± 22.88 mg/g of ascorbic acid at the same concentration. Furthermore, the total antioxidant capacity of ACCF-liposomes and ACCF was found to be 861.62 ± 20.01 and 825.32 ± 32.10 mg/g ascorbic acid, respectively at high concentration. This is indicated that the total antioxidant capacity of ACCF-liposomes and ACCF was concentration dependent as it increased with an increase in their concentration (Fig. 6a).

##### Free radical scavenging potency

ACCF and ACCF-liposomes have substantial free radical scavenging potency in a concentration-dependent manner (Fig. 6b&c). Furthermore, the liposomal formulation of ACCF potentiates its free radical scavenging potency since it can scavenge DPPH and NO radicals to some extent comparable to ACCF and ascorbic acid (standard antioxidant) at all concentrations. Regarding DPPH scavenging potency, ACCF-liposomes can scavenge 73.5 ± 0.52% of DPPH at a concentration of 50 μg/ml, but ACCF scavenged only 56.63 ± 2.76% at the same concentration. Furthermore, ACCF-liposomes exhibited high antioxidant potency with a higher percentage (95.24 ± 0.83%) approximately to ascorbic acid (standard antioxidant), which scavenges DPPH by 94.7 ± 0.40% at a concentration of 400 μg/ml (Fig. 6b). Additionally, the ACCF-liposomes showed greater inhibition of NO formation compared to ACCF, which had minimal inhibition at all concentrations. At high concentrations, both ACCF and ACCF-liposomes were found to provide greater inhibition than ascorbic acid, which typically inhibits NO formation by 86.75 ± 0.01%. However, ACCF and ACCF-liposomes were able to inhibit NO formation by 95.49 ± 0.60% and 97.34 ± 0.01%, respectively, as demonstrated in Fig. 6c. Regarding IC_50_ values, the DPPH assay results showed that the IC_50_ values for ACCF, ACCF-liposomes, and ascorbic acid were 38.75, 23.53, and 18.75 µg/ml, respectively (Table 4). Meanwhile, the IC_50_ values for the same compounds in the NO assay were 38.46, 26.82, and 27.77 µg/ml. These findings validate that the liposomal formulation of ACCF enhances its ability to scavenge free radicals.

**Table 4:** Half maximal inhibitory concentration (IC_50_) values of antioxidant and anti-inflammatory potencies

Sample	IC_50_ value (µg/ml)		
	**Antioxidant potency**		**Anti-inflammatory potency**
	**DPPH assay**	**Nitric oxide assay**	
ACCF	38.75	38.46	41.66
ACCF-liposomes	23.53	26.82	31.45
Ascorbic acid	18.75	27.77	–
Aspirin	–	–	37.03

*ACCF Allolobophora caliginosa* coelomic fluid, *DPPH* 1,1-Diphenyl-2-picrylhydrazyl

##### Anti-inflammatory potency of ACCF and ACCF-liposomes

**Fig. 7:**
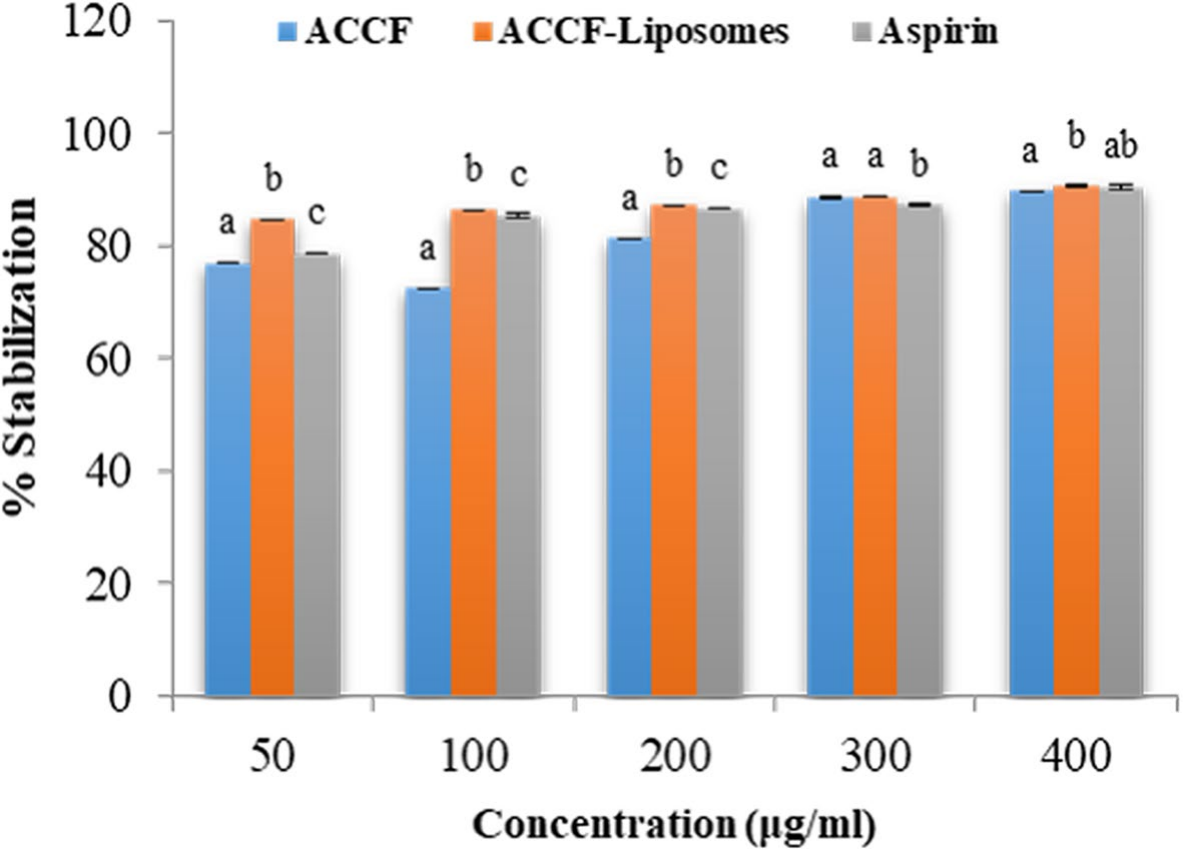
Stabilization percentage of HRBC membrane by ACCF, ACCF-liposomes, and Aspirin demonstrating anti-inflammatory potency. Values are the mean of three replicate determinations ± SEM. Values with different superscript letters are significantly different (P < 0.05) for each concentration. ACCF: *Allolobophora caliginosa* coelomic fluid. HRBC: human red blood cell

ACCF and ACCF-liposomes effectively stabilized the HRBCs membrane as compared to aspirin in a concentration-dependent manner, confirming remarkable anti-inflammatory effect via their ability to resist the lysis of the HRBC membrane (Fig. 7). At a concentration of 50 µg/ml, a pronounced the HRBC membrane stabilization occurred at 84.57 ± 0.057% for ACCF-liposomes. In contrast, ACCF and aspirin exhibited 76.77 ± 0.02% and 78.58 ± 0.13% HRBC membrane stabilization, respectively (Fig. 7). Figure 7 also illustrated that ACCF-liposomes showed a pronounced anti-inflammatory potency comparable to that of ACCF at all concentrations ranged from 50 to 400 µg/ml, indicating liposomal formulation potentiates its anti-inflammatory activity. Furthermore, the IC_50_ of ACCF and ACCF-liposomes were found to be 41.66 and 31.45µg/ml, respectively, but aspirin showed an IC_50_ of 37.03 µg/ml, suggesting that ACCF-liposomes could be utilized as a potent anti-inflammatory agent (Table 4).

##### Fibrinolytic potency of ACCF and ACCF-liposomes

**Fig. 8:**
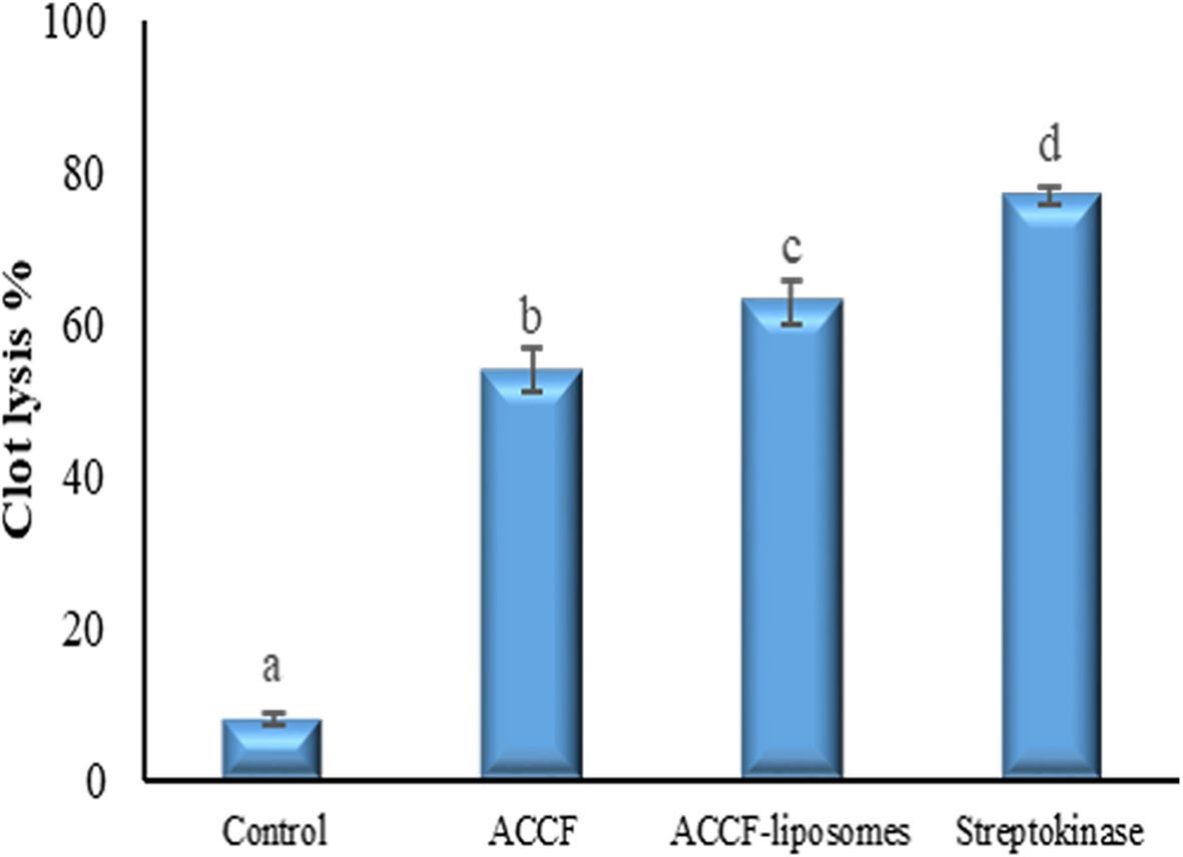
Fibrinolytic potency of ACCF, ACCF-liposomes, and streptokinase assessed by Clot lysis percentage. Values are the mean of three replicate determinations ± SEM. Values with different superscript letters are significantly different (P < 0.05). ACCF: *Allolobophora caliginosa* coelomic fluid

As demonstrated in Fig. 8, the liposomal formulation of ACCF had the most effective thrombolytic activity with 62.95 ± 2.88% clot lysis rate compared with standard streptokinase, which showed clot lysis of 76.62 ± 1.15%. On the other hand, ACCF had a 53.89 ± 2.90% of clot lysis rate. Meanwhile, distilled water, a negative control, showed only negligible clot lysis of 7.93 ± 0.66% after 90 min incubation with clot.

## Discussion

Currently, encapsulation technologies become competitive tools in developing novel antioxidant compounds utilized as active constituents in the food and medicinal industries. These technologies can protect the active ingredients, enhance their stability, and prolong their release in the gastrointestinal tract [37]. Among encapsulation techniques, liposome is attractive for the food and medical industries because it provides apparent advantages, especially their biodegradability, biocompatibility, and ability to encapsulate a wide range of bioactive compounds [38]. Therefore, the present study was intended to develop a liposomal formulation for *Allolobophora caliginosa* coelomic fluid (ACCF) as a natural antioxidant compound to improve its stability, enhance its release during gastrointestinal tract and potentiate its biological activities such as antioxidant, anti-inflammatory, and fibrinolytic.

For the effective applications of liposomal formulation, the stability, efficacy, and the in vitro behavior of prepared ACCF-liposomes must be determined via various physical attributes. Particle size is one of the most important physical attributes to characterize liposomal formulation. Particle size is an imperative physicochemical attribute of liposome distribution and cellular uptake because it influences their absorption efficiency, especially in the gastrointestinal tract and the endocytosis-dependent cellular uptake [12, 39]. Jiang et al. [40] reported that the reduced liposomal particle size leads to the high surface area and stability of colloidal systems, promoting the accumulation of entrapped active compounds at the target site and extending their half-life in the blood. As presented in the current study, the average hydrodynamic size of the prepared ACCF-liposomes was 98 nm confirming the nanosized property of ACCF-liposomes and their distinct physicochemical properties. The attained nano-size of ACCF-liposomes affords promising liposomal stability and high release of its entrapped ACCF, as Naghavi et al. [41] stated. Also, Naghavi et al. [41] suggested that the small size of the liposomal formulation confirmed the higher cohesion and packing among the polar chains in the vesicular membrane of the liposomal system. Thus, the tissue distribution and cellular uptake of ACCF-liposomes will be potentiated, improving its efficiency as a food-grade and pharmaceutical-grade product. Small-size of the prepared ACCF-liposomes may be accredited to phenolic compounds of ACCF, as Rafiee et al. [42] reported. They demonstrated that the entrapment of phenolic compounds in the liposomal system could reduce the liposome size, probably via the interaction between the hydroxyl group of phenolic compounds and lipid acyl chains, and effectively inducing conformational changes in liposomal bilayer structures, resulting in a decrease of the liposomal size [42]. Moreover, Bochicchio et al. [43] attributed the nanoscale size of the prepared liposomes to a distinct mechanical power impact of ultrasound action on liposome vesicles, considerably reducing the particle size and improving the uniformity of the size distribution. This finding was generally similar to the results of Liu et al. [28] and Gülseren and Corredig [44], who prepared the tea polyphenol liposomes with average hydrodynamic sizes of 160 nm and about 90 nm, respectively.

Furthermore, the nanosized ACCF-liposomes were substantiated by the TEM and XRD analysis, which displayed a homogenous and spherical shape with an average size of 40 nm and 21.62 nm, respectively, suggesting that the ACCF-liposomes achieved the desired nanoscale size. The difference between the particle size recorded through TEM, XRD, and DLS studies may be attributed to a difference in sample processing during size measurement [45]. Accordingly, DLS is considered more desirable for particle size analysis comparable to the TEM study since nanomaterials used mainly for nutritional and biomedical applications are suspended in an aqueous medium rather than used as a powder [46]. Additionally, the present TEM study confirmed the bilayer structure of ACCF-liposomes, indicating a well-organized assembly of the liposome phospholipids, as Liu et al. [47] demonstrated.

Regarding adequate drug delivery, the PDI can detect the tendency of lipid nanocarriers to accumulate in the target tissue by determining the homogeneity degree of the liposomal formulation [39]. A PDI of 0.3 and below reflects adequate drug delivery to target tissue and indicates a homogenous population of phospholipid vesicles [48]. This study revealed that the PDI value of ACCF-liposomes equals 0.29, indicating a monodisperse vesicular system and promising nutritional and clinical applications. The present finding is in harmony with the reports of Marín et al. [49] and Mohammadi et al. [50].

The zeta potential is an essential indicator of liposomal physical stability in the colloidal system by determining liposomal particles' surface electrical charges [51]. In the current study, the zeta potential of prepared ACCF-liposomes was -38.66 mV, indicating extreme stability of ACCF-liposomes and their aggregation resistance. This finding was agreed with Savagheb et al. [51], who reported that liposomal formulations with a high zeta potential, usually higher than 30 mV or less than –30 mV, are highly electrostatic stable because the particles will repel each other and decrease the tendency of aggregation as well as enhance the dispersability in the aqueous media. Furthermore, the obtained negative value of the zeta potential may be accredited to the fatty acids of liposomal formulation that possess anionic surface charge on liposomes, as Katherine et al. [52] stated. Additionally, the high zeta potential value is related to the negatively charged carboxyl group of phenolic acids [53], and phenolic acids, particularly gallic acid have been reported as one of the most important phenolic compounds in ACCF [8]. Thus, the present study attributed the high zeta potential of prepared ACCF-liposomes to its phenolic acids. This attribution was supported by the previous research of Rashidinejad et al. [54], who demonstrated a high zeta potential value of liposomes-entrapped green tea phenolic compounds. Furthermore, the current result regarding the zeta potential of ACCF-liposomes is comparable with previous studies on encapsulating different natural antioxidants in the liposomal system [37, 51].

Concerning effective liposomal entrapment, the FT-IR analysis is used to verify the successful liposomal entrapment of ACCF and to monitor possible changes in the structure of the liposomal lipid and active compound [55]. The current FT-IR study demonstrated that the encapsulation of ACCF within the formulated liposomes did not develop any new bonds, as confirmed by the FT-IR spectrum of liposomes entrapped ACCF did not contain any additional absorption bands when compared with ACCF and free liposome spectra. These results are compatible with the former study conducted by Konda et al. [56] clarified that no appearance or disappearance of absorption peaks in their doxorubicin -lipid liposomal formulation was detected, confirming the absence of any covalent linkage between the doxorubicin and lipid membrane. But the present study showed few differences between absorption peaks of ACCF-liposomes and unloaded liposomes, probably due to the overlap of ACCF functional groups with strong bands of free liposomes, as Ben-Fadhel et al. [57] suggested. Additionally, the obtained FT-IR spectrum confirmed the interaction of ACCF bioactive compounds with fatty acids chain or choline polar head in liposome vesicles as indicated by reduced absorption peaks of ACCF-liposomes related to CH and CH_3_ stretching modes, CH_2_ blending mode (1519.65 cm^−1^), C-O ester stretch (1639.22 cm^−1^). These changes in FT-IR spectra suggested slight primary structural changes between free liposomes and ACCF-loaded liposomes. These findings are supported by a previous study of Ben-Fadhel et al. [57], who demonstrated slight primary structural changes in liposomes after being loaded with citrus extract.

The efficacy of colloidal delivery systems relies on their ability to proficiently encapsulate, maintain, stabilize, and release bioactive compounds [58]. Entrapment efficiency is one of the most significant physicochemical indicators for assessing liposomal system effectiveness in the nutraceutical and medical industries [59]. Remarkably, the current study exhibited that ACCF effectively entrapped within liposomes with relatively high entrapment efficiency percent (77.58%), subsequently signifying an effective encapsulation delivery system in the band as the bioavailability of entrapped ACCF as Basiri et al. [60] reported. This finding was strengthened by the nano size of ACCF-liposomes that provides a large surface area for the encapsulation of ACCF as Homayoonfal et al. [61] revealed that nano-liposomes cause an increased volume of the aqueous nucleus and culminating in enhanced entrapment efficiency percent of hydrophilic bioactive compounds. Drvenica et al. [37] reported a similar entrapment efficiency percent for encapsulating sinigrin (62%) into liposomes. Furthermore, an entrapment efficiency of 50.20% was obtained for encapsulating phenolic compounds of algal extract in soy lecithin liposomes, which agrees with the present result [62]. The slight disparity may be related to a difference in the encapsulation method or lecithin concentration applied, as Savaghebi et al. [62] suggested. The relatively high entrapment efficiency percent is primarily allied to ACCF concentration since with the elevation of ACCF concentration, the chance of electrostatic interaction between the ACCF and negatively charged liposomal phospholipids increases, and subsequently, the tremendous amount of ACCF entrapped in the liposomal bilayer [61]. Furthermore, the high entrapment efficiency percent may be attributed to lecithin used in liposome preparation. Takahashi et al. [63] reported that increases in lecithin concentration by up to 10% promote high encapsulation efficiencies of hydrophobic molecules. Additionally, Fang et al. [64] clarified that several factors, especially proper cholesterol and lipid ratios, increase entrapment efficiency percent and liposomal stability.

The performance of entrapped bioactive compounds in drug delivery systems depends on their release behavior [65]. The in vitro drug release is a crucial index to evaluate liposomes' targeted and controlled release characteristics. Generally, the bioactive molecules entrapped within liposomes are released mainly through three mechanisms which are 1) dispersion of molecules via the intact liposomal membrane into the surrounding environment, 2) corrosion of liposomal membrane induced by phospholipid degradation, and 3) swelling of pores in the liposomal membrane permitting the leakage of entrapped bioactive molecules [66]. In the current study, the release behavior of liposomes entrapped ACCF showed an initial burst release of 25% within 2h of diffusion. Then, the sustained ACCF release up to 70% was noticed in the following 2 to 8 h, indicating that the release of entrapped ACCF from the liposomal structure may be via diffusion and certain changes in liposomal membranes like erosion or swelling. Shashidhar and Manoha [67] attributed the initial fast release rate typical to bioactive compound detachment from the liposomal surface. But the advanced sustained release may be due to the active compound being mainly entrapped inside the bilayer lipid structure of the liposomes, so the entrapped compound diffused firstly from the inner layer to the surface and then to releasing medium [65]. Moreover, liposome's controlled release behavior may be attributed to ACCF incorporated into the lipid bilayer, which prevents ACCF from rapidly dispersing into the medium, as Pan et al.[68] stated. Additionally, the obtained results contribute new insights into the stability of the liposomal structures, mainly probably due to the interaction between ACCF and the phospholipid and, accordingly, the preceding slow release of the active compound [29]. Hence, the entrapment of ACCF in liposomes could effectively provide ACCF constantly in the body and prolonged residence time in circulation, facilitating bioactive compounds to reach target sites. These results are supported by previous studies of Shashidhar and Manoha [67] and Drvenica et al. [37].

Storage stability, including physical and chemical stability, is an essential index of any drug delivery system. Zhou et al. [69] demonstrated that physical instability could lead to entrapped bioactive material leakage and the cluster or fusion of vesicles. Thus, the assessment of the physicochemical properties of liposomes during storage is convenient for determining their physical stability. The physical stability result revealed that the prepared ACCF-liposomes are more likely colloidal stable as indicated by a non-significant change in their mean hydrodynamic diameter, zeta potential, and PDI as well as entrapment efficiency percent after storage at 4°C for one month, revealing adequate electrostatic stabilization of the prepared ACCF-liposomes and this finding was confirmed by the obtained negative value of zeta potential. The stability of ACCF-liposomes is attributed to the uniform distribution of charge density around their surface, accomplishing a thermodynamically stabilized state [67]. Furthermore, the lipid bilayer structure and fluidity are controlled by the medium's pH, so the current study evaluated the stability of ACCF- liposomes at different pH. The ACCF-liposomes are highly stable at pH 4.5, 6.8, and 7.4, comparable to their stability at pH 1.5, implying their stability in biological fluids at physiological pH. On the other hand, the relatively low stability of ACCF-liposomes at pH 1.5 may be due to acidic pH reducing the surface charge of liposomes and decreasing the repulsion forces between them, subsequently increasing the size of the vesicles and the tendency to aggregation [62]. Thus, the integrity of the phospholipid bilayers is reduced, and the release of entrapped material is increased [70]. Whereas the higher stability of ACCF-liposomes at pH 4.5, 6.8, and 7.4 is attributed to the maximum retention of surfactant molecules on the surfaces of the liposomes and, accordingly, arresting aggregation. These results are supported by the previous study by Shashidhar and Manohar [67].

During encapsulation, liposomes can protect the encapsulated bioactive compounds from environmental conditions and provide a restrained release of the entrapped material [71]. Permeability is one of the main parameters involving environmental protection and controlled release characteristics of liposomes. The permeability rate is the main index to indicate liposomal stability and to demonstrate the circumstances of entrapped bioactive compounds in liposomes during storage [71]. In this study, the permeability rate was 3.20% after 30 days of storage at 4 °C, indicating the stability of prepared ACCF-liposomes during storage at low temperature. The obtained low value of permeability rate is directly related to a low concentration of ACCF-liposomes, which promotes decreasing the negative charges and repulsive forces and hence reduces the permeability rate, and this resulted in stability of the liposomal structure as Naghavi et al. [41] demonstrated. Additionally, the permeability rate depends on the method of liposome preparation, where the permeability rate decreased by increasing process time to provide an appropriate opportunity to import phosphatidylcholine molecules to the liposome structure and hence improve stability [71].

Interestingly, liposomes offer a novel approach to transporting and delivering natural antioxidants to the gastrointestinal system. The oral route is the most suitable drug administration route, especially during long-term treatment [13]. Thus, the ACCF-liposome’s behavior during digestion was evaluated using an in vitro simulated gastrointestinal system. In this study, the release of ACCF from liposomal formulation increased gradually with the increase of digestion time during in vitro gastric and intestinal digestion process, revealing the sustained release behavior of ACCF and enhancing its bioaccessibility, especially in intestinal digestion. But the release of ACCF was still less than 35% at the end of simulated gastric digestion. The current study attributes this primarily to the retained integrity of liposomal structures after simulated gastric digestion, which has been ascertained in the earlier study [72]. Liu et al. [47] revealed that pepsin could not hydrolyze and damage the phospholipid membrane of the liposomes during simulated gastric digestion, resulting in retaining entrapped materials during liposomal formulation. Furthermore, the current study clarified that ACCF-liposomes are highly stable in intestinal digestion conditions comparable to their stability in gastric digestion, and this is indicated by 67.25% of ACCF was retained in the liposomes during simulated gastric digestion process but over 93% of ACCF was still retained in the liposomes during simulated intestinal digestion process after 120 min. These findings are ascertained by the current result of ACCF-liposomes stability at different pH. Relatively excessive ACCF release during simulated gastric digestion may be attributed to the acidic pH of simulated gastric fluid that can induce bridging, flocculation, and droplet aggregation [73]. Moreover, the relatively high ionic strength in the simulated gastric juice could diminish electrostatic interaction in the suspensions, developing droplet aggregation, as Liu et al. [74] demonstrated. Meanwhile, the high stability of ACCF-liposomes during simulated intestinal digestion may be related to the changes in pH, and the presence of bile salts could enhance the electrostatic interaction between droplets and prevent their aggregation [75]. Overall, the findings imply that the liposomal formulation containing ACCF improved its bioaccessibility and may lead to higher bioavailability and intestinal uptake because a high portion of ACCF remains entrapped. Accordingly, it is accessible for further release and transformation to a highly bioactive form. These results are reinforced by the previous studies of Drvenica et al. [37] and Uribe et al. [76].

One of the most imperative aims of nanoencapsulation is to preserve and potentiate the biological activities of entrapped bioactive compounds during their development and storage [77]. Consequently, the ongoing study appraised various bioactivities of free and nano-liposomal ACCF. Generally, oxidative stress and inflammation associated with the cellular overproduction of ROS and pro-inflammatory mediators can initiate and develop chronic degenerative diseases by activating the redox-responsive signaling cascades, severely damaging cellular compartments [78]. Regarding antioxidant potency, the present study showed that ACCF and ACCF-liposomes possess a potent free radical scavenging activity on DPPH and NO radicals compared to ascorbic acid as a standard antioxidant. The free radical scavenging potency of ACCF is highly correlated to its phenolic and flavonoid compounds, particularly gallic acid and quercetin, which are found in abundant quantities in ACCF, as Sadek et al.[8] recommended. Besides phenolic and flavonoids compound, the potent antioxidant potency of ACCF is attributed to its hydrophobic amino acid group, especially alanine, valine, leucine, and isoleucine, which can donate its proton to bind with the DPPH^•^ radical, and this hypothesis is strengthened by a previous study [8]. Moreover, the antioxidant activity of ACCF was significantly enhanced after nano-liposome encapsulation. ACCF-liposomes exhibited a considerable antioxidant potency comparable to that of ACCF, as indicated by the substantial total antioxidant capacity of ACCF-liposomes equivalent to 807.67 mg/g ascorbic acid. In harmony with the present results, Noudoost et al. [59] confirmed that nano-liposome encapsulation technology improved green tea extract's free radical scavenging potency against DPPH radicals by increasing extract dispersability in the environment. Additionally, Zokti et al.[79] reported that encapsulated green tea extract exhibited a higher antioxidant activity than free green tea extract. The relatively high antioxidant potency of ACCF-liposomes may be due to the great amount of phenolic compounds of ACCF entrapped during liposomal formulation. This postulation is supported by Noudoost et al. [59]. Also, Beg et al. [80] revealed that liposomal encapsulation induced chemical modification to antioxidant structure, improving antioxidants' pharmacological and pharmacokinetic properties. The enhanced antioxidant action of ACCF during liposomal encapsulation may be due to unique physicochemical characteristics of the liposomal structure and its bioactivity, as Sułkowski et al.[81] demonstrated.

Interestingly, the current study also revealed that ACCF and ACCF-liposomes effectively stabilized the HRBCs membrane, analogous to the lysosomal membrane, reinforcing remarkable anti-inflammatory potency via their ability to resist the lysis of the HRBC membrane. The ability of ACCF to stabilize the HRBC membrane may be attributed to its binding with the HRBC membrane and subsequently altered surface charge of the cells, resulting in counteracting physical interaction or promoting the dispersion by related repulsion of identical charges [8, 82]. Also, the current investigation attributed the anti-inflammatory potency of ACCF to its gallic acid, quercetin and rutin content which either likely restrain the activity of cyclooxygenase (COX) and lipoxygenase or inhibit discharge lysosomal constituents by stabilizing the lysosomal membrane as recently described by Sadek et al. [8]. Again, Sadek et al. [8] implied the stabilizing potency of ACCF to its amino acid constituent, especially glycine and histidine. Furthermore, liposomal encapsulation enhanced the anti-inflammatory potency of ACCF because the encapsulation process could improve or increase the activity of bioactive molecules by protecting them from the environment and controlling their release [83]. Moreover, the relatively anti-inflammatory potency of ACCF-liposomes is attributed to their small size that can interact efficiently with the HRBCs membrane. The cellular uptake of liposomes is a binary-step manner involving the adsorption or attachment of liposomes onto the cell surface and following endocytosis or content release [84]. Also, the relatively high entrapment efficiency percent of ACCF-liposomes is directly related to their promising anti-inflammatory potency. This is because increasing the ACCF concentration potentiates its cellular uptake and increases its ability to stabilize the HRBCs membrane [85]. This result was similar to the earlier study of Shariare et al. [86] demonstrated that phytol is a potent anti-inflammatory agent, and this activity was enhanced significantly after liposomal encapsulation.

Furthermore, liposomal encapsulation offers promising noninvasive and instant thrombolytic treatment approaches that may help reduce cardiovascular disorders' morbidity and mortality [87]. Hence, the ongoing investigation evaluated the thrombolytic potency in vitro of free and liposomal ACCF to appraise the use of ACCF-liposomes as an alternative approach to cardiovascular disease management. In the present study, the liposomal formulation of ACCF exhibited the highest thrombolytic activity followed by ACCF when compared with standard streptokinase. The current investigation mainly attributed the fibrinolytic potency of ACCF to its fibrinolytic enzymes, especially serine proteases [88]. Serine proteases is one of the most powerful fibrinolytic enzymes contributing to stimulated fibrinolysis and plasminogen which enhances the transformation of insoluble fibrin clot into dissolved fibrin, resulting in effective thrombosis process [89]. Also, the thrombolytic potency of ACCF is may relate to its flavonoid content, particularly quercetin, as Mitra et al. [90] recommended. Concerning the thrombolytic potency of the liposomal formulation, the notable fibrinolytic impact of ACCF-liposomes is attributed to their remarkable entrapment efficiency of 77.58%, resulting in the sustained release of ACCF [91]. Overall, the present study recommends that liposomal formulation potentiate various biological activities of ACCF so that it is an appropriate candidate for improving the delivery of ACCF.

## Conclusion

Liposomes are a promising drug delivery system for natural antioxidants in the food and medical industries. They can improve their chemical stability, bioavailability, and health-promoting impact by facilitating intracellular delivery and increasing the retention period of entrapped antioxidants inside the cell. In the current study, *Allolobophora caliginosa* coelomic fluid (ACCF) was effectively encapsulated into lecithin-based liposomes in nanometric size with homogeneous dispersion (average hydrodynamic size 98 nm and spherical shape). Additionally, the ongoing study proved that liposomal encapsulation offers an excellent tool to improve the behavior release and the stability of ACCF under simulated gastrointestinal conditions. Regarding the biological activities of ACCF, the liposomal formulation potentiates its biological activities, such as antioxidant and anti-inflammatory activities, as well as fibrinolytic potency comparable to that of its free form. Therefore, the current results offer great potential for encapsulating valuable compounds relevant to nutraceutical, functional food, and pharmaceutical applications. Nevertheless, an advanced investigation must be performed to evaluate the effectiveness of liposomal formulation for ACCF in vivo and its assembly probability in the future.

## Data Availability

The datasets of the current study are available from the corresponding author on a reasonable request.

## Abbreviations

ACCF: *Allolobophora caliginosa* Coelomic fluid
DPPH: 1,1 Diphenyl-2-picrylhydrazyl
DLS: Dynamic light scattering
PDI: Polydispersity index
TEM: Transmission electron microscope
FT-IR: Fourier transform infrared
XRD: X- ray diffraction
PBS: Phosphate buffer saline
SGF: Simulated gastric fluid
SIF: Simulated intestinal fluid
IC_50_: Half maximal inhibitory concentration
NO: Nitric oxide
HRBC: Human red blood cell
